# Athlete Body Image and Eating Disorders: A Systematic Review of Their Association and Influencing Factors

**DOI:** 10.3390/nu16162686

**Published:** 2024-08-13

**Authors:** Qingqing Li, Hansen Li, Guodong Zhang, Yang Cao, Yun Li

**Affiliations:** 1 College of Physical Education, Southwest University, Chongqing 400715, Chinalygd777@swu.edu.cn (G.Z.); 2 International College, Krirk University, Bangkok 10220, Thailand; 3 Clinical Epidemiology and Biostatistics, School of Medical Sciences, Faculty of Medicine and Health, Orebro University, 70182 Orebro, Sweden; yang.cao@oru.se; 4 Unit of Integrative Epidemiology, Institute of Environmental Medicine, Karolinska Institutet, 17177 Stockholm, Sweden

**Keywords:** athletes, body image, dietary imbalance, systematic review

## Abstract

Body image and eating disorders pose significant challenges to the overall health of athletes. However, divergent findings exist regarding the potential association between athletes’ body image and eating disorders. This systematic review aims to examine the relationship between these two variables and identify the modifiers of the association, such as gender, age, race, and exercise type. A search was conducted in five databases (Web of Science, PubMed, APA PsycINFO, ProQuest, and EBSCO), aiming to identify studies on athletes and involved body image and eating disorders in their conclusions. Ultimately, thirty-one studies were included for systematic evaluation. The results of the studies indicate that the relationship between athletes’ body image and eating disorders is complex and inconclusive. In some types of sports, eating disorders can occur even when athletes are satisfied with their body image. Furthermore, female athletes, particularly young female athletes, and athletes involved in sports associated with leanness are more prone to eating disorders and body dissatisfaction. Due to limited resources in this type of research, there is a lack of comprehensive inclusivity across sports disciplines, genders, races, and levels of sports proficiency, which warrants further research.

## 1. Introduction

Athletes are a high-risk group for eating disorders, with a high prevalence of eating disorder attitudes and behaviors estimated between 14% and 45% [1,2]. Previous research has suggested that individuals with higher levels of body dissatisfaction are more likely to show eating disorder behaviors [3,4,5], such as dieting, unhealthy eating, and weight control practices [6,7]. Specifically, eating disorder behaviors (such as dieting and weight control) can serve as means to achieve weight loss and ultimately attain a desired body image. Compared to non-athletes, athletes have a higher prevalence of eating disorders, ranging from subclinical symptoms to clinical diagnosis [8]. Previous systematic reviews have identified gymnasts as having a higher risk of eating disorders, with one of the main reasons being distorted and dissatisfied body image [9]. While research has identified body dissatisfaction as a cause of eating disorders [10] and that athletes with eating disorders are more likely to experience body dissatisfaction [11], it remains unclear whether there are other underlying factors associated with body image and eating disorders.

According to Stice (1994)’s dual-pathway model, when individuals perceive that their personal appearance does not align with the ideal, the internalization of appearance-related ideals can lead to body dissatisfaction [12]. Body dissatisfaction can directly lead to eating disorders or indirectly cause negative outcomes such as weight loss attempts through dietary restriction, which increases the risk of binge eating and purging [13]. In the general population, there is evidence that body dissatisfaction is associated with an increased risk of eating disorders [14]. Interestingly, there is inconsistent evidence regarding whether body dissatisfaction can predict eating disorders in athletes. Compared to non-athletes, athletes may be more susceptible to the influence of other underlying factors, thereby affecting the relationship between body dissatisfaction and eating disorders.

It is worth noting that the association between athletes’ body image and eating disorders may be influenced by factors such as gender, age, race, sports discipline, and sports proficiency. For example, studies suggest that female athletes may be more prone to body dissatisfaction and eating disorders compared to male athletes [15]. Adolescent female athletes may be more susceptible to eating disorders and body dissatisfaction than adult female athletes [16]. The prevalence of body image and eating disorders may vary among athletes in different sports disciplines [17,18,19]. Some studies have implied that body image and eating disorders may vary among athletes of different races and sports proficiency levels [20,21]. Furthermore, eating disorders appear to be more prevalent in sports that emphasize specific body weight or leanness, as compared to more general sports [18]. Similar cases have also been reported when it comes to the comparisons between aesthetic sports and heavyweight sports [22]. Even with these indications, there is no systematic review specifically focusing on the potential influencers.

Despite over 20 years of research on athletes’ body image and eating disorders, previous systematic reviews have only focused on either eating disorders or body image, as well as the factors influencing either of the two variables [23,24]. The association between athletes’ body image and eating disorders has been largely overlooked, let alone the influencers of the association. To provide researchers and professionals with a better understanding of this topic, we conducted this study with the aim of compiling relevant evidence to explore the association between athletes’ body image and eating disorders, as well as their potential influencers. These efforts may contribute to the replication of future studies and facilitate better control of external variables to obtain more reliable findings.

## 2. Materials and Methods

This study was conducted in accordance with the Preferred Reporting Items for Systematic Reviews and Meta-Analyses (PRISMA) guidelines for guidance [25]. Prior to the main literature review, a scoping search was conducted on 10 May 2024, using Google Scholar and Web of Science to determine whether there was sufficient literature on athletes’ body image and eating disorders worthy of such a review. After consulting PROSPERO (International Prospective Register of Systematic Reviews), it was confirmed that there were no registered systematic reviews on this topic. The present review has now been registered with PROSPERO (No. CRD42024503403).

### 2.1. Searching Strategy

The literature search was conducted by the first author on 5 July 2024. The main search strategy involved using the terms “athletes”, “body image”, and “eating disorders” to retrieve records with titles, abstracts, and keywords from five online databases (PubMed, APA PsycINFO, Web of Science, EBSCO, and ProQuest Central). The search process for all five databases was identical, and there were no date restrictions. The search terms (Table 1) were based on previous systematic reviews using search terms related to body image in adult males [26], body image in the context of eating disorders and muscle dysmorphia [27], and athletes (MeSH and free terms). Only articles in English were considered in this study. The complete electronic search strategy is provided in the Supplementary Materials.

### 2.2. Eligibility Criteria

Our eligibility criteria obeyed the PICOS principle as follows [28]:

- P (population): participants who are defined as athletes;
- I (intervention): any interventions;
- C (comparison): any comparisons;
- O (outcomes): any outcomes related to eating disorders and body image;
- S (study design): observational designs and any other designs that possibly report disorders and body image (even if it was only part of descriptive statistics).

Notably, when identifying athlete populations, we adhere to the principles established in previous studies and only considered traditional sports events [29]. This led to the inclusion of bodybuilding athletes while excluding individuals involved in chess and esports [30]. Additionally, our study included controlled trials, cross-sectional, and longitudinal studies to deeply understand the potential associations between variables. The study had no time limitations and covered athletes of all age ranges, with a particular focus on adolescent and adult athletes. We considered and included all types of eating disorders, such as anorexia nervosa, bulimia nervosa, and binge eating disorder.

### 2.3. Data Extraction

The data from the included studies were independently extracted by two reviewers (Q.Q.L. and H.S.L.) and thoroughly reviewed by a third reviewer to resolve any potential discrepancies (G.D.Z.). Data extraction from the selected articles was conducted by the first author (Q.Q.L.). The following data were collected from the eligible studies: study authors, publication year, sample size, sample characteristics, gender distribution, whether it involved aesthetic sports, research methods, measurement of body image and/or eating behaviors, and study findings.

### 2.4. Quality Assessment

The risk of bias in the full-text articles included in this analysis was assessed independently by two reviewers (Q.Q.L. and H.S.L.) using a 12-item quality assessment tool [31,32]. In case of disagreement, a third reviewer (G.D.Z.) reviewed and resolved the discrepancies. This assessment was aimed to gauge the potential bias in the studies included. The checklist encompassed various criteria such as sample description, adequacy of statistical analyses, and completeness of results reporting. Each criterion was rated as “yes”, “partial”, or “no”. A rating of “yes” corresponded to a score of “2”, “partial” to a score of “1”, and “no” to a score of “0”. Each article received a total score, with a maximum of 24 points achievable. Articles scoring between 24 and 17 were classified as high quality, those scoring between 16 and 9 as moderate quality, and those scoring between 8 and 0 as low quality. A detailed breakdown of the quality assessment for each study is available in Appendix A.

### 2.5. Data Process

To fully understand the relationship between eating disorders and body image, we considered both clinical diagnosis results and general results measured by questionnaires. Although all studies included in this review are quantitative, there is considerable heterogeneity concerning indicators and assessment tools. These differences eliminated the need for quantitative integration of the results. Therefore, following the advice of others, we have used non-qualitative methods to extract and summarize key findings cautiously and draw conclusions accordingly [33].

## 3. Results

### 3.1. Study Selection

Two authors (Q.Q.L. and H.S.L.) independently screened the articles obtained through the search following the established inclusion criteria. Any potential disagreements were resolved by a third reviewer (G.D.Z.). Initially, duplicate studies were removed. Then, the titles and abstracts of the articles were assessed, followed by a review of the full texts of the studies that met the initial criteria.

**Figure 1:**
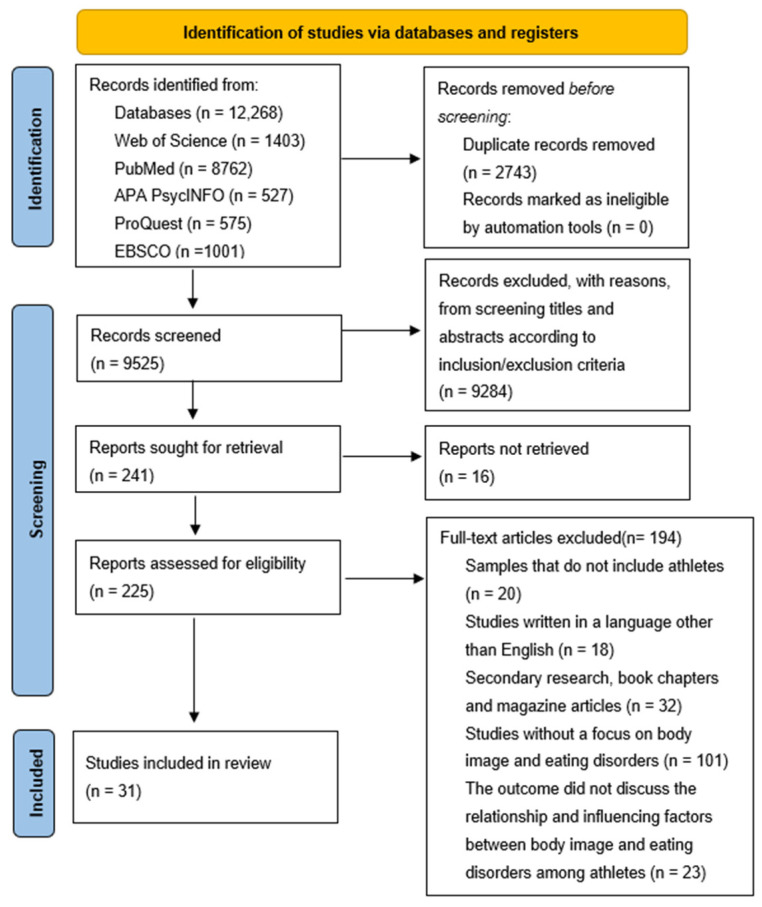
PRISMA flow diagram for the selection process.

Figure 1 illustrates the research selection process based on the PRISMA guidelines. A total of 12,268 studies were generated from the database search, and after removing duplicates, 9525 studies were retained for further evaluation. Through screening titles and abstracts based on inclusion/exclusion criteria, 9284 articles were excluded, leaving 225 studies that met the criteria for full-text screening. Among them, 194 studies were excluded for not meeting the inclusion/exclusion criteria, resulting in the final inclusion of 31 studies (Figure 1).

### 3.2. Study Characteristics

The study included a total of 31 articles (study characteristics and main findings are shown in Table 2). Among them, 15 articles employed a cross-sectional design, providing an instantaneous understanding of athletes’ body image and dietary behaviors at a specific point in time. Fourteen articles were controlled trials [16,17,18,19,34,35,36,37,38,39,40,41,42,43], allowing for a more comprehensive exploration of the associations between athletes’ body image and eating disorders. One article was a mixed-methods study [44], combining quantitative and qualitative research methods to comprehensively reveal body image and eating disorders among male artistic gymnasts. Another article was a longitudinal study conducted over a four-month timeframe [45], focusing on male college athletes.

The study included a total of 11,184 participants. Publication dates ranged from 1996 to 2023, with five studies involving NCAA participants [20,46,47,48,49]. Thirteen studies exclusively recruited females [17,18,21,35,37,38,41,46,47,50,51,52,53], nine studies exclusively recruited males [19,34,39,44,45,48,49,54,55], and the remaining nine studies included both females and males [15,16,20,36,40,42,43,56,57]. Seven studies were conducted among aesthetic athletes, such as gymnastics and dance. The study results were obtained using scales that measure body image satisfaction and the extent of eating disorders. In the included studies, 25 different scales were used to measure body image satisfaction, and 11 scales were used to measure the extent of eating disorder behaviors, with the most commonly used scale being the Eating Attitudes Test-26 (EAT-26).

**Table 2:** Key characteristics of included studies.

Category	Study	Design	Sample (*n*)	Female %	Aesthetic Sports %	Body Image Measure (Interpretation)	Eating Disorder Measure (Interpretation)	Key Findings	Quality
Studies evaluating the effect of body image on eating disorders	de Bruin, Oudejans [37]	Controlled trials	68 gymnasts and 85 schoolgirls (n = 153)	100%	44.4%	Body Image Questionnaire, BIQ (-)	Six items of the Bulimia Test-Revised, BULIT-R (-)	Both elite and non-elite gymnasts have a positive body image, but frequent dieting behavior still exist.	High
	Cusack, Petrie [45]	Longitudinal analysis	Male collegiate athletes (n = 452)	0%	1.5%	Use the five items as individual indicators on the latent variable of body satisfaction (-)	Eating Disorder Examination Questionnaire Short, EDEQ-S (higher scores indicate higher levels of eating disordered symptomatology)	Body satisfaction is a major antecedent of eating disorders in male athletes.	High
	Satterfield and Stutts [49]	Cross-section	NCAA male wrestler (n = 103)	0%	0%	Body Parts Satisfaction Scale for Men, BPSS–M (higher scores indicate more satisfaction with body image)	Eating Pathology Symptoms Inventory, EPSI (higher scores indicate more severe eating disorders)	Male wrestlers exhibit relatively high body satisfaction; however, they engage in numerous unhealthy weight- loss behavior in their quest for muscle development.	High
	Pallotto, Sockol [47]	Cross-section	Female Division I college athletes (n = 212)	100%	3.25%	Body Image Satisfaction Scale, BISS (higher scores indicate more dissatisfaction with body image)	Eating Attitudes Test, EAT-26 (higher scores indicate more severe eating disorders)	Weight pressure from parents, peers, and the media is directly associated with body dissatisfaction and indirectly linked to eating disorders through the internalization of thinness and muscular ideals.	High
	Petrie and Moore [48]	Cross-section	Male collegiate athletes (n = 1975)	0%	1.37%	Body Parts Satisfaction Scales, BPSS (higher scores indicate more satisfaction with body image)	Eating Disorder Examination Questionnaire, EDEQS (higher scores indicate more severe eating disorders)	Greater athlete satisfaction with their current weight, shape, and thinness during the COVID-19 transition was associated with a reduction in eating disorders.	High
	Paixao, Oliveira [50]	Cross-section	Portuguese young female athletes (n = 142)	100%	100%	Cognitive Fusion Questionnaire Body Image, CFQ-BI, and Perfectionistic Self-Presentation Scale-Body Image, PSPS-BI (higher scores indicate greater level of self- presentation)	Eating Disorder Examination, EDE-Q (higher values indicating higher severity of eating psychopathology)	In aesthetic athletic girls, preoccupation with body image-related thoughts may be associated with the desire to present an idealized body image to others, potentially resulting in eating disorders attitudes and behavior.	Moderate
	Gibson, Hindle [54]	Cross-section	Elite rugby union players in New Zealand (n = 26)	0%	0%	-	EDI-3 include DT, bulimia, and body dissatisfaction	Although rugby players are considered by society to have the “ideal” tall muscular physique, they are still at increased risk of body dissatisfaction and eating disorders when they face sports-related stress.	Moderate
	Voelker, Gould [53]	Cross-section	Five US states female figure skaters (n = 272)	100%	100%	Contour Drawing Rating Scale, CDRS (-)	Eating Attitudes Test, EAT-26 (-)	General body dissatisfaction in athletes is a significant predictor of eating disorders.	High
	Anderson, Petrie [46]	Cross-section	280 NCAA, Division-I, female collegiate gymnasts, and 134 swimmers and divers (n = 414)	100%	67.6%	Body Satisfaction (higher scores indicate more satisfaction)	The 9-item Dietary Intent Scale, DIS, and 10-item Dutch Restrained Eating Scale, DRES (higher scores indicated more restraint in eating behavior)	The negative mood, body dissatisfaction, and dietary restriction of athletes were directly related to symptoms of bulimia.	High
	Francisco, Narciso [40]	Controlled trials	Portugal adolescents (n = 725)	62.5%	33.8%	Contour Drawing Rating Scale, CDRS (-)	Eating Disorder Examination-Questionnaire, EDE-Q (-)	Body image was the strongest predictor of eating disorders.	High
Studies evaluating the effect of eating disorders on body image	Neves, Meireles [42]	Controlled trials	40 elite athletes, 245 non-elite athletes and 128 non-athlete adolescents (n = 413)	73.6%	100%	Body Shape Questionnaire, BSQ (the higher the total score, the greater the body dissatisfaction)	Eating Attitudes Test-26, EAT-26 (the higher the score, the higher the risk of eating disorders)	In the arts program, among elite, non-elite, and non-athletes, risk behaviors for eating disorders were the factor most strongly associated with body dissatisfaction.	High
	Rosendahl, Bormann [43]	Controlled trials	576 young German athletes and 291 non-athletes (n = 867)	43.9%	(-)	Body image and body ideal were measured with male and female silhouettes representing different weight categories (-)	Eating Attitude Test, EAT-26 (EAT 0–9 are defined as subjects with normal eating behaviors and attitudes, and EAT > 10 as subjects with eating disorders behavior and Attitudes)	Athletes who had dieting experiences reported dissatisfaction with their body shape and weight more frequently than those who did not, as well as eating disorder behaviors and attitudes.	High
	de Bruin, Oudejans [38]	Controlled trials	19 athletics with eating disorders and 33 athletics without eating disorders (n = 52)	100%	100%	Contextual body image questionnaire for athletes, CBIQA Visual Analogue Scales, VAS Body-Self Relations Questionnaire, MBSRQ (-)	Eating disorder examination questionnaire, EDE-Q (-)	On a daily basis, athletes with eating disorders were more negative about their appearance than athletes without eating disorders.	High
	Michou and Costarelli [41]	Controlled trials	74 Greek Female basketball players and 80 nonathletes female (n = 154)	100%	0%	Body- Self-Relations Questionnaire, MBSRQ (-)	Eating Attitudes Test, EAT-26 (-)	There were no significant differences in DE attitudes between female basketball players and non-players; however, DE attitudes in the whole group of women (n = 154) were significantly positively associated with anxiety levels and unhealthy body image.	High
	Costarelli and Stamou [36]	Controlled trials	20 taekwondo and judoka athletes and 40 non-athletes (n = 60)	76.7%	0%	Multidimensional Body-Self Relations Questionnaire, MBSRQ (-)	Eating Attitudes Test, EAT-26 (-)	In comparison to non-athletes, athletes exhibited higher emotional intelligence and healthier body image, with no significant differences in attitudes toward eating disorders.	High
	Goltz, Stenzel [55]	Cross-section	52 competing in weight-class sports, 52 in sports with aesthetic ideals, and 52 competing in athletics, swimming, triathlon, and horse racing (n = 156)	0%	33.3%	Body Shape Questionnaire, BSQ (the score range is used to determine body image)	Eating Attitudes Test, EAT-26 and Bulimic Investigatory Test Edinburgh, BITE (the score interval determines whether there is an eating disorder)	Nearly a quarter of the athletes displayed eating disorder, associated with dissatisfaction with their body image. Athletes with higher body fat were more likely to experience dissatisfaction with their body image. No significant variations in eating behavior and body image were observed among athletes in different sports categories.	High
	Salbach, Klinkowski [52]	Cross-section	50 elite rhythmic gymnasts, 58 female patients with AN, and 56 high school girls (n = 164)	100%	100%	The Test for Detecting Body Image Distortion in Children and Adolescents (-)	EDI-2 (-)	People with anorexia nervosa are more likely to have a distorted body image than elite rhythmic gymnasts and high school students.	High
Studies stratified by gender	Pinto, Dolan [44]	A mixed method	Elite male artistic gymnastics (n = 17)	0%	100%	Muscle Silhouette Measure, MSM, and Male Body Checking Questionnaire, MBCQ (higher scores indicative of a higher frequency of body checking behaviors)	Eating Attitude Test, EAT-26, Bulimic Investigatory Test, BITE, and Binge Eating Scale, BES (0–17 points overeating, 18–36 moderate symptoms, 27–46 represents severe)	Eating disorders are not common among male elite gymnasts.	Moderate
	Blackmer, Searight [56]	Cross-section	Varsity athletes at a midwestern university (n = 103)	54.4%	(-)	Body Uneasiness Test, BUT (-)	Eating Attitudes Test, EAT-26 (-)	Female athletes scored significantly higher than male athletes on all measures related to eating disorders and body image	High
	Milligan and Pritchard [57]	Cross-section	American Western Athletic Conference (n = 176)	56%	8.5%	Body Shape Questionnaire, BSQ (higher scores indicating greater body dissatisfaction)	Eating Attitude Test, EAT-26 (20 points cut-off value to decide)	The order in which eating disorder behaviors were predicted was body dissatisfaction, self-esteem, and types of sports (thin or non-thin), while for men, the order in which eating disorder behaviors were predicted was body dissatisfaction.	Moderate
	Kristjánsdóttir, Sigurðardóttir [15]	Cross-section	43 aesthetic athletes, 116 endurance athletes, 76 weight-class athletes, 140 fitness athletes, and 380 ball athletes (n = 755)	33.9%	5.7%	Body Shape Questionnaire, BSQ (higher score indicates greater level of body dissatisfaction)	Bulimia Test-Revised, BULIT-R and Eating Disorder Examination Questionnaire, EDE-Q (higher score reflects a greater eating-related pathology)	Female athletes exhibit higher scores in body image concerns compared with their male counterparts, and they are more susceptible to eating disorders than male athletes.	High
	Salbach, Klinkowski [52]	Cross-section	50 elite rhythmic gymnasts, 58 female patients with AN, and 56 high school girls (n = 164)	100%	100%	The Test for Detecting Body Image Distortion in Children and Adolescents (-)	EDI-2 (-)	People with anorexia nervosa are more likely to have a distorted body image than elite rhythmic gymnasts and high school students.	High
Studies stratified by age	Borowiec, Banio-Krajnik [35]	Controlled trials	Polish female adolescents (12–18 years) and adult athletes (19–30 years) (n = 241)	100%	23.2%	Feelings and Attitudes Toward Body Scale—by Orbach and Mikulincer (higher scores indicate more satisfaction with body image)	The Eating Attitudes Test, EAT-26 (higher scores indicate more severe eating disorders)	Compared with adult athletes, the risk of eating disorders in adolescent athletes was significantly linked to body satisfaction and the types of sports. Among adolescent athletes, the risk of eating disorders was most closely associated with participating in lean, non-aesthetic sports.	High
	Baceviciene, Jankauskiene [16]	Controlled trials	Lithuanian athletes (n = 1003)	51.3%	13.56%	Multidimensional Self-Relations Questionnaire Appearance Scale, MBSRQ-AS and Body Appreciation Scale 2, BAS-2 (-)	Eating Disorder Examination Questionnaire 6, EDE-Q 6 (higher score indicates more frequent DE behaviors, more severe weight and size problems)	Adolescent female athletes exhibit a higher susceptibility to body dissatisfaction and eating disorders compared with adult female athletes. In contrast, adult male athletes experience more frequent eating disorders and concerns about being overweight compared with adolescent male athletes.	High
Studies stratified by race	Johnson, Crosby [20]	Cross-section	NCAA (n = 1445)	38.9%	-	Include in EDI-2	Eating disorders inventory, EDI-2 (these subscales range from 0 to 21 for drive and for thinness, and from 0 to 27 for body dissatisfaction)	White female athletes were more likely to report body dissatisfaction and eating disorder behaviors than black female and male athletes.	High
Studies stratified by sports events	Kong and Harris [18]	Controlled trials	Australian elites and recreational sporting organizations and clubs (n = 320)	100%	54.4%	Figure Rating Scale, FRS (-)	Eating Attitudes Test, EAT-26 (-)	Athletes from leanness-focused sports reported higher levels of body dissatisfaction and greater symptoms of eating disorders than those with non-leanness-focused sports.	High
	Reinking and Alexander [51]	Cross-section	84 collegiate athletes and 62 non athletes (n = 146)	100%	-	Include in EDI-2	Eating disorders inventory, EDI-2 (-)	Lean-sport athletes had a higher risk of body dissatisfaction and eating disorders compared with athletes in non-lean sports.	High
	Goldfield [17]	Controlled trials	20 competitive female bodybuilders and 25 recreational female weight training athletes (n = 45)	100%	44.4%	Six-item Drive for Bulk scale was developed as a modification of the Body Dissatisfaction scale of the EDI (-)	Eating Disorder Inventory, EDI (-)	In contrast to female athletes engaged in recreational weight training, competitive bodybuilders reported significantly higher incidences of overeating, excessive focus on weight or body shape, strict dieting, and strenuous exercise to control their weight.	High
	Pietrowsky and Straub [19]	Controlled trials	48 male athletes and 32 non-athletes (n = 80)	0%	0%	Body Image Assessment, BIA (-)	Fragebogen zum Essverhalten, FEV (scores more than 7 indicate a high degree of restrained eating behavior, and less than 4 points on that scale were classified as unrestrained eaters)	Adult lightweight rowers scored highly on eating restraint compared with heavy-weight rowers, and reported higher levels of body dissatisfaction when hungry than when full.	High
	Firoozjah, Shahrbanian [39]	Controlled trials	Iranian male adolescent athletes (n = 124)	0%	0%	Body-esteem scale for adolescents and adults, BESAA (higher scores indicate more positive body-esteem)	Eating attitudes test-26, EAT-26 (clinical cut-off score of ≥20 indicates eating disorders)	During COVID-19, individual athletes are more likely to suffer from eating disorders and distorted body image than team athletes.	High
	Anderson, Zager [34]	Controlled trials	68 male recreational and competitive bodybuilders and 50 non bodybuilders (n = 118)	0%	57.6%	Include in EDI-2	Eating Disorders Inventory, EDI-2 (-)	Bodybuilders had higher body satisfaction than non-bodybuilders, but no difference was found in the risk of eating disorders.	High
	Goltz, Stenzel [55]	Cross-section	52 competing in weight-class sports, 52 in sports with aesthetic ideals, and 52 competing in athletics, swimming, triathlon, and horse racing (n = 156)	0%	33.3%	Body Shape Questionnaire, BSQ (the score range is used to determine body image)	Eating Attitudes Test, EAT-26 and Bulimic Investigatory Test Edinburgh, BITE (the score interval determines whether there is an eating disorder)	Nearly a quarter of the athletes displayed an eating disorder associated with dissatisfaction with their body image. Athletes with higher body fat were more likely to experience dissatisfaction with their body image. No significant variations in eating behavior and body image were observed among athletes in different sports categories.	High
	Smith, Gay [21]	Cross-section	American Cheerleaders (n = 268)	100%	100%	Sex-Specific Figural Stimuli Silhouette, SIL (-)	Eating Attitudes Test, EAT-26 (-)	Cheerleaders are at risk for eating disorders and dissatisfied body image regardless of sporting level.	High

Note. The study by Goltz, Stenzel [55] belongs to both the studies evaluating the effect of eating disorders on body image and studies stratified by sports events. The study by Salbach, Klinkowski [52] belongs to both the studies evaluating the effect of eating disorders on body image and studies stratified by gender.

### 3.3. Assessment of Quality

According to the quality assessment tool used in this review, all articles were considered to have at least moderate quality (for more detailed information on the quality of included studies, please refer to Appendix A). Four studies were assessed as having moderate quality [44,50,54,57]. The primary reason was the lack of description of participant characteristics and reasons for excluding non-participants. Additionally, 17 studies did not have a control group, which may introduce some uncertainty in inferring the relationship between body image and dietary disturbances. The included studies thoroughly discussed their results, contributing to a better understanding of the limitations of the research and the substantive content of the findings.

### 3.4. The Relationship between Eating Disorders and Body Image

#### 3.4.1. Potential Effect of Athletes’ Body Image on Eating Disorders

Ten studies investigated the impact of athletes’ body image on eating disorders [37,40,45,46,47,48,49,50,53,54]. Among them, two studies found that even when athletes were satisfied with their body image, they still exhibited eating disorder behaviors [37,49]. For instance, elite and non-elite gymnasts had positive body image, but still engaged in frequent restrictive eating behaviors [37]. Additionally, Satterfield, Noah A., and Stutts, Lauren A. investigated body satisfaction and eating disorders in wrestlers, finding that wrestlers had relatively high body satisfaction but still engaged in unhealthy weight-loss behaviors due to a strong pursuit of muscularity [49]. The pressure from weight expectations led athletes to internalize unrealistic body ideals, ultimately resulting in body dissatisfaction and eating disorders. Pallotto et al. (2022) found that general weight pressures from parents, peers, and the media were indirectly associated with eating disorders through the internalization of thin and muscular ideals [47].

A cross-sectional study conducted during the COVID-19 transition period indicated that athletes’ higher satisfaction with current weight, body shape, and thinness was associated with a reduction in eating disorders [48]. Worsened athlete body image was associated with fluctuations in body composition (e.g., weight gain, muscle loss), changes in their relationship with food (e.g., perceived lack of control), and an increase in symptoms of eating disorders [58]. The pandemic caused by COVID-19 may induce stress in athletes and could potentially exacerbate the risk of eating disorders [48]. Furthermore, a cross-sectional study found that among aesthetic sportswomen, the entanglement of thoughts related to body image might be associated with the need to present a perfect body image to others, leading to disruptions of eating attitudes and behaviors [50]. This study emphasized the relevance of body image-related processes in the correlation with eating disorders [50]. Another cross-sectional investigation of elite male rugby players’ body image and the risk of eating disorders found that despite being considered socially “ideal” for their tall, muscular physique, rugby players still faced an increased risk of body dissatisfaction and eating disorders under sport-related pressure [54].

Furthermore, four studies indicate that athletes’ body image is a predictive factor for eating disorders [40,45,46,53]. Voelker et al. (2014) examined the association between eating disorders and body dissatisfaction in female figure skaters, suggesting that attention to weight and appearance, along with dissatisfaction with one’s body, might be crucial tools for preventing and detecting eating disorders in female figure skaters [53]. Similarly, Anderson et al. (2011) investigated college gymnasts, swimmers, and divers, finding that negative emotions, body dissatisfaction, and dietary restrictions were directly related to symptoms of binge eating disorder in female athletes [46]. Another study found that body satisfaction was a major precursor to eating disorders in male athletes [45]. A controlled study also found that, among elite athletes, the strongest predictor of eating disorders was dissatisfaction with body image [40].

#### 3.4.2. Potential Effect of Eating Disorders on Athletes’ Body Image

Existing research has demonstrated a connection between eating disorders and dissatisfaction with athletes’ body image [36,38,41,42,43,52,55]. Specifically, Neves et al. (2016) investigated elite athletes, non-elite athletes, and non-athletes in artistic disciplines, finding that their eating disorder behaviors were most closely associated with body dissatisfaction [42]. Moreover, studies have found that nearly one-fourth of male athletes exhibit eating disorder behaviors, which are linked to dissatisfaction with body image [55]. Similarly, two additional studies have shown that athletes with eating disorders are more likely to experience body dissatisfaction compared to those without eating disorders, both among high school athletes [43] and college or adult athletes [38]. The research also investigated differences in body image dimensions in the daily lives of athletes with and without eating disorders, revealing that athletes with eating disorders have a more negative perception of their appearance in everyday life [38].

Among the seven studies focusing on the impact of eating disorders on athletes’ body image, three explored the relationship between eating disorders and body image in athletes and non-athletes [36,41,42]. For instance, Salbach et al. (2007) found that elite artistic gymnasts, compared to female anorexia nervosa patients and high school girls, did not exhibit significant issues in attitudes towards eating disorders but showed slight distortions in body image [52]. Additionally, female basketball players and non-athlete women did not show significant differences in attitudes towards eating disorders, but attitudes towards eating disorders in the entire group of women (n = 154) were significantly positively correlated with anxiety levels and unhealthy body image [41]. Similarly, another study investigated body image, emotional intelligence, anxiety levels, and attitudes towards eating disorders in taekwondo and judo athletes (experimental group) and non-athletes (control group). The study found no significant differences in attitudes toward eating disorders between the experimental and control groups. Regression analysis revealed a significant positive correlation between attitudes towards eating disorders and anxiety levels and self-classified body weight related to body image [36].

### 3.5. Potential Influencers of the Association

Based on the literature found in the search, we have also compiled some potential confounding or moderating factors in the association between “body image—eating disorders”.

#### 3.5.1. Role of Gender

Four quantitative studies found that female athletes are more prone to eating disorders and body dissatisfaction than male athletes [15,44,56,57]. For example, the predictive sequence of eating disorder behaviors in females includes body dissatisfaction, self-esteem, and the type of sport (thin or non-thin). In contrast, for males, the predictive sequence of eating disorder behaviors is primarily associated with body dissatisfaction [57]. Regression analysis revealed that body dissatisfaction is the only variable related to eating disorder behaviors in male athletes [57]. Additionally, a cross-sectional study focusing on gender difference found that female athletes scored higher than males [15]. Similarly, another cross-sectional study found that compared to male athletes, females reported higher levels of eating disorder behaviors and body image distress [56].

A mixed-methods study indicated that eating disorder phenomena are not widespread among elite male artistic gymnasts [44]. However, female gymnasts experience more internal and external pressure to maintain low body weight [44]. For instance, a female gymnast may excel during competitions but may face comparisons with other female athletes or public figures on social media. If her body shape does not align with the ideal standards portrayed on social media, she may feel pressure, triggering self-doubt and dissatisfaction with her appearance.

Taken together, these studies provide preliminary evidence supporting the notion that female athletes are more susceptible to eating disorders [59], which may further lead to dissatisfaction with body image.

#### 3.5.2. Role of Age

Two controlled studies examined the impact of athlete age on body image and eating disorders [16,35], aiming to compare differences in body image and eating disorders among athletes in different age groups. Baceviciene et al. (2023) found that compared to adult female athletes, adolescent female athletes are more prone to body dissatisfaction and eating disorders. Conversely, for male athletes, the situation is reversed, with adult male athletes experiencing more eating disorders and concerns about their bodies than adolescent males [16]. In addition, Borowiec et al. (2023) found that teenage female athletes are more likely to experience eating disorders than adult female athletes, and the risk of eating disorders in adolescent female athletes is related to the type of sport and body satisfaction [35]. In summary, both studies indicate that adolescent female athletes are more susceptible to body dissatisfaction and eating disorders than adult female athletes.

#### 3.5.3. Role of Race

A cross-sectional study examined racial differences in self-esteem and various eating attitudes and behaviors among elite college athletes [20]. Through surveys of athletes from 11 different sports across 11 schools, the study found that compared to black female and male athletes, white female athletes are more prone to body dissatisfaction and eating behavior disorders [20]. This study suggests a new perspective that white female athletes may be more susceptible to body dissatisfaction and eating disorders than black athletes. However, it is important to note that this conclusion lacks additional supporting evidence as only one study indicated this, and further research is needed to confirm these findings.

#### 3.5.4. The Role of Sports Events

Existing evidence indicates that athletes from different sports may exhibit varying degrees of association between body image and eating disorders. Specifically, two studies suggest that female athletes emphasizing leanness are more prone to body dissatisfaction and eating disorders compared to non-thin female athletes [18,51]. These sports emphasizing a thin physique and slender appearance may lead athletes to adopt extreme dietary measures to meet specific appearance standards. Additionally, a controlled study found that competitive bodybuilders reported significantly higher rates of binge eating, excessive weight or shape concern, strict dieting, and intense exercise to control weight compared to recreational weight-training female athletes [17]. Appearance and body shape evaluation play a crucial role in competitive bodybuilding, potentially leading athletes to adopt extreme dietary measures to meet strict aesthetic standards [60]. Another study found that adult lightweight rowers are more susceptible to body dissatisfaction and eating disorders than heavyweight rowers [19]. Athletes in lightweight rowing may be influenced by weight restrictions imposed by the category, requiring them to maintain weight within specified lightweight ranges for competitions, leading to stricter dietary measures [61].

During COVID-19, a controlled study found that individual athletes are more prone to eating disorders and distorted body image compared to team athletes. However, no differences were observed in the dieting and oral control subscales [39]. Additionally, Anderson et al. (1996) studied male bodybuilders and found that, compared to non-bodybuilding male athletes, male bodybuilders had higher body satisfaction but no difference in the risk of eating disorders [34]. Similarly, in another cross-sectional study, the authors stratified their analysis by level of competition, but there was no evidence that different levels of impact changed [21].

Generally, the majority of included studies support the notion that athletes in sports emphasizing leanness are more likely to experience body dissatisfaction and eating disorders.

## 4. Discussion

Body dissatisfaction and eating disorders are common psychological health issues among athletes [11,62,63]. This study is presented to explore the association between athletes’ body image and eating disorders and identify the modifiers of the association. Overall, we found a complex relationship between athletes’ body image and eating disorders, which cannot be definitively concluded at present. External factors such as gender, age, race, exercise level, and type of sport may influence these associations and need further consideration in future research.

### 4.1. The Association between Eating Disorders and Body Image

The association between athletes’ body image and eating disorders is not yet conclusive. On one hand, some studies suggest that there is no connection between athletes’ body image and eating disorders [37,47,49,54]. The eating disorder behaviors of athletes may be driven by other mechanisms, potentially involving factors such as sports performance, extreme pursuit of ‘clean’ eating, appearance expectations, and individual definitions of success. For instance, female athletes who are satisfied with their appearance may believe that adjusting their weight and appearance can enhance their athletic performance [37], leading to extreme dietary behaviors. Furthermore, for individuals with orthorexia nervosa, obsession with healthy eating and exercise addiction may intertwine [64,65]. On the other hand, there is research supporting the association between athletes’ body image and eating disorders [40,45,46,53]. Specifically, for some athletes, body dissatisfaction may be considered a predictive factor for eating disorders [66]. When athletes pursue ideal body types or appearance standards, they may adopt varying degrees of dietary control, especially in sports that emphasize appearance.

### 4.2. Influencers of the “Body Image—Eating Disorders” Association

#### 4.2.1. Gender

The studies we included generally support the impact of gender on body image and eating disorders, with females appearing to be more sensitive to both psychological issues. This phenomenon may involve various factors, with social and cultural pressures playing a significant role [67,68]. The standards imposed by society on the appearance and body image of women are often more stringent, leading to increased vulnerability to body image insecurity among females. Simultaneously, societal expectations of women’s roles may contribute to their heightened concern about weight and appearance [69]. This societal pressure may trigger eating disorders.

#### 4.2.2. Age

The studies included in this review suggest that adolescent female athletes are more likely to experience body dissatisfaction and eating disorders compared to adult female athletes [16,35]. Adult female athletes, in contrast to adolescents, exhibit more positive body image [16] and demonstrate greater resilience in the face of societal and cultural pressures to conform to stereotypical body ideals [70]. Adult female athletes may no longer face appearance and weight-related pressures from parents, while adolescents may experience more psychological violence and neglect related to weight from their parents [71]. Peers, especially teammates, also play a crucial role as sources of appearance and weight pressure for adolescent athletes [72]. In addition, coaches and uniforms also exert pressure on athletes regarding weight, body shape, and dietary behaviors [73].

#### 4.2.3. Sports Events

The studies we included suggest that the types of sports may influence body dissatisfaction and eating disorders. Studies have shown that athletes in sports emphasizing leanness are more prone to experiencing body dissatisfaction and eating disorders [17,18,19,34,51,59]. These studies highlight the specific pressures that sports focused on body shape and leanness may exert on athletes. Athletes may face excessive scrutiny of their body shape and appearance while pursuing their sporting goals, thereby increasing the risk of body dissatisfaction and eating disorders.

### 4.3. Limitations

Firstly, among the 31 studies included, 11 different scales were used to measure eating disorders, and 25 scales were used to measure body image. Due to significant heterogeneity in research designs and the measurements of included studies, a meta-analysis of the key findings was not performed, which forbids a comprehensive understanding of the form and strength of the association between athletes’ body image and eating disorders. Therefore, a future quantitative review is expected when more relevant studies emerge.

Secondly, as our included studies only involved some types of sports and athletes of certain characteristics, our findings may not be generalized to all contexts or all athletic populations. Future research should include various sports and athletes and consider a wider age range and gender diversity.

Thirdly, we discussed the factors that might influence the “body image-eating disorders” association on the basis of included studies. These studies primarily used grouping surveys or analysis methods to explore the relationships between these external factors and either body image or eating disorders. The methods employed by these studies might be somewhat rough, considering these influencing factors but not controlling for a broader range of variables. Consequently, the strength of the evidence from these results is not strong. Based on these findings, we hypothesized that several factors, such as gender and age, that could influence this association might be more accurately described as “confounding factors” because they may simultaneously affect both body image and eating disorders. However, we should recognize that factors such as gender are often considered as moderators in observational studies as well. Nevertheless, since no studies have simultaneously studied the “body image-eating disorders” association while considering the moderating effects of these external factors, we cannot directly conclude to treat these variables as moderators. Despite this, we call for future research to consider both potential roles (as confounders and moderators) of these variables and use different statistical methods to ensure the robustness of the findings.

## 5. Conclusions

This study was conducted to assess the association between athletes’ body image and eating disorders as well as related modifiers. We found that the evidence concerning the association was inconclusive. Additionally, we collected some factors that may influence this association, playing roles as moderators or confounding factors. While empirical evidence on factors such as athlete level and ethnicity are still relatively scarce, future research should consider these factors and aim for more reliable conclusions in observational studies. Additionally, other potential moderating factors such as mental health status, social support, and cultural background should also be considered.

## Figures and Tables

**Table 1:** Search terms.

Terms	Search Terms
Term A	“Athlete” OR “Professional Athletes” OR “Athlete, Professional” OR “Athletes, Professional” OR “Professional Athlete” OR “Elite Athletes” OR “Athlete, Elite” OR “Athletes, Elite” OR “Elite Athlete” OR “College Athletes” OR “Athlete, College” OR “Athletes, College” OR “College Athlete”
Term B	“Body image” OR “body dissatisfaction” OR “body satisfaction” OR “body esteem” OR “body appreciation” OR “appearance” OR “body functionality” OR “body preoccupation” OR “body shame” OR “body awareness” OR “body anxiety” OR “interocept *” OR “shape concern” OR “shape dissatisfaction” OR “weight concern” OR “weight dissatisfaction”
Term C	“eating disorder *” OR “disordered eat *” OR “diet *” OR “bing *” OR “purg *” OR “anorex *” OR “bulim *” OR “bigorex *” OR “muscle dysmorp *” OR “adonis complex *” OR “manorex *”

* Note. Term A and Term B and Term C were combined using the ‘AND’ operator.

## Data Availability

The data used during the current study are available from the corresponding author upon reasonable request.

## Supplementary Materials

The following supporting information can be downloaded at: https://www.mdpi.com/article/10.3390/nu16162686/s1**.**

## Appendix A

**Table A1:** Quality appraisal of included studies.

Authors										
Criteria—Quantitative Studies	Smith et al. (2022) [21]	Firoozjah et al. (2022) [39]	Neves et al. (2016) [42]	Voelker et al. (2014) [53]	Francisco et al. (2013) [40]	Blackmer et al. (2011) [56]	Anderson et al. (2011) [46]	Reinking and Ale xander (2005) [51]	Johnson et al. (2004) [20]	Anderson et al. (1996) [34]
1. Clearly stated aims	Y	Y	Y	Y	Y	Y	Y	Y	Y	Y
2. Participant eligibility and recruitment strategy clearly documented	Y	Y	Y	Y	N	P	N	Y	Y	Y
3. Main features of population/design described	Y	Y	P	Y	Y	Y	Y	Y	Y	Y
4. Non-responders (and non-participants) described	Y	Y	N	N	N	N	N	Y	N	N
5. Presence of a control group	N	Y	Y	N	Y	N	N	N	N	Y
6. Main limitations identified and acceptable	Y	Y	P	Y	Y	Y	Y	Y	Y	N
7. Sample size justified	Y	Y	N	N	Y	N	Y	N	Y	Y
8. No evidence of selective reporting of results	Y	Y	Y	Y	Y	Y	Y	Y	Y	Y
9. Statistical methods described	Y	Y	Y	Y	Y	Y	Y	Y	Y	Y
10. Statistical methods appropriate	Y	Y	Y	Y	Y	Y	Y	Y	Y	Y
11. Measures relevant, validated and described adequately	Y	Y	Y	Y	Y	Y	Y	Y	Y	P
12. Results discussed adequately	Y	Y	Y	Y	Y	Y	Y	Y	Y	P
Total Score	22	24	18	18	20	17	18	22	20	18

Score Key: Y (Yes) = Score of 2; P (Partial) = Score of 1; N (No) = Score of 0; and DK (Don’t know) = Score of 0. Scoring: 17–24 = good quality; 9–16 = acceptable quality; and 0–8 = low quality.

**Table A2:** Quality appraisal of included studies.

	Authors										
Criteria—Quantitative Studies	Petrie & Moore (2023) [48]	Borowiec et al. (2023) [35]	Baceviciene et al. (2023) [16]	Pallotto et al. (2022) [47]	Cusack et al. (2022) [45]	Satterfield and Stutts (2021) [49]	Paixao et al. (2021) [50]	Pinto et al. (2020) [44]	Kristjánsdóttir et al. (2019) [15]	Gibson et al. (2019) [54]	Goltz et al. (2013) [55]
1. Clearly stated aims	Y	Y	Y	Y	Y	Y	Y	Y	Y	Y	Y
2. Participant eligibility and recruitment strategy clearly documented	P	Y	Y	Y	Y	N	N	Y	Y	N	Y
3. Main features of population/design described	Y	Y	Y	Y	Y	Y	Y	P	Y	Y	Y
4. Non-responders (and non-participants) described	N	N	Y	N	N	N	N	N	N	N	Y
5. Presence of a control group	N	Y	Y	N	N	N	N	N	N	N	N
6. Main limitations identified and acceptable	N	Y	Y	Y	Y	Y	Y	N	Y	N	P
7. Sample size justified	Y	Y	Y	Y	Y	P	N	N	Y	Y	Y
8. No evidence of selective reporting of results	Y	Y	Y	Y	Y	Y	Y	Y	P	Y	Y
9. Statistical methods described	Y	Y	Y	Y	Y	Y	Y	Y	Y	Y	Y
10. Statistical methods appropriate	Y	Y	Y	Y	Y	Y	Y	Y	Y	Y	Y
11. Measures relevant, validated and described adequately	Y	Y	Y	Y	Y	Y	Y	P	Y	P	Y
12. Results discussed adequately	Y	Y	Y	Y	Y	Y	Y	Y	Y	Y	Y
Total Score	17	22	24	20	20	17	16	14	19	15	21

Score Key: Y (Yes) = Score of 2; P (Partial) = Score of 1; N (No) = Score of 0; and DK (Don’t know) = Score of 0. Scoring: 17–24 = good quality; 9–16 = acceptable quality; and 0–8 = low quality.

**Table A3:** Quality appraisal of included studies.

Authors										
Criteria—Quantitative Studies	Salbach et al. (2007) [52]	Milligan and Pritchard (2006) [57]	Kong and Harris (2015) [18]	Michou and Costarelli (2011) [41]	de Bruin et al. (2011) [38]	Rosendahl et al. (2009) [43]	Goldfield (2009) [17]	Costarelli and Stamou (2009) [36]	Pietrowsky and Straub (2008) [19]	de Bruin et al. (2007) [37]
1. Clearly stated aims	P	Y	Y	Y	Y	Y	Y	Y	Y	Y
2. Participant eligibility and recruitment strategy clearly documented	Y	N	Y	P	N	Y	P	Y	P	p
3. Main features of population/design described	Y	Y	Y	Y	P	Y	Y	Y	N	Y
4. Non-responders (and non-participants) described	N	N	Y	Y	N	N	N	N	N	N
5. Presence of a control group	N	N	Y	Y	Y	Y	Y	Y	Y	Y
6. Main limitations identified and acceptable	Y	Y	Y	P	Y	P	Y	P	N	N
7. Sample size justified	Y	N	Y	N	N	N	N	N	Y	Y
8. No evidence of selective reporting of results	Y	Y	Y	Y	Y	Y	Y	Y	Y	Y
9. Statistical methods described	Y	Y	Y	Y	Y	Y	Y	Y	Y	Y
10. Statistical methods appropriate	Y	Y	Y	Y	Y	Y	Y	Y	Y	Y
11. Measures relevant, validated and described adequately	Y	Y	Y	Y	Y	Y	Y	Y	Y	Y
12. Results discussed adequately	Y	Y	Y	Y	Y	Y	Y	Y	Y	Y
Total Score	19	16	24	20	17	19	19	19	17	19

Score Key: Y (Yes) = Score of 2; P (Partial) = Score of 1; N (No) = Score of 0; and DK (Don’t know) = Score of 0. Scoring: 17–24 = good quality; 9–16 = acceptable quality; and 0–8 = low quality.
