# Supplementary material for: Athlete Body Image and Eating Disorders: A Systematic Review of Their Association and Influencing Factors
Source: Nutrients. 2024 Aug 13;16(16):2686. doi: 10.3390/nu16162686 (PMC11356870; doi:10.3390/nu16162686)
Supplement: Supplementary file 1 [file nutrients-16-02686-s001.zip › nutrients-3117429-supplementary.pdf]

Full electronic search strategy for Web of Science, PubMed, APA PsycINFO, ProQuest, and EBSCO databases：

(“eating disorder*” OR “disordered eat*” OR “diet*” OR “bing*” OR “purg*” OR “anorex*” OR “bulim*” OR “bigorex*” OR “muscle dysmorp*” OR “adonis complex*” OR “manorex*”) AND (“Body image” OR “body dissatisfaction” OR “body satisfaction” OR “body esteem” OR “body appreciation” OR “appearance” OR “body functionality” OR “body preoccupation” OR “body shame” OR “body awareness” OR “body anxiety” OR “interocept*” OR “shape concern” OR “shape dissatisfaction” OR “weight concern” OR “weight dissatisfaction”) AND(“Athlete” OR “Professional Athletes” OR “Athlete, Professional” OR “Athletes, Professional” OR “Professional Athlete” OR “Elite Athletes” OR “Athlete, Elite” OR “Athletes, Elite” OR “Elite Athlete” OR “College Athletes” OR “Athlete, College” OR “Athletes, College” OR “College Athlete”)
